# A Conversation with Karina Sand

**DOI:** 10.1021/acscentsci.3c01574

**Published:** 2023-12-22

**Authors:** Carolyn Wilke

In 2022, researchers shared that they had uncovered an ancient ecosystem from sediments in the Kap København Formation in Greenland. By sequencing DNA left behind in the environment, they obtained a snapshot of a long-lost forest that hosted mastodons, hares, reindeer, and geese. The genetic material sealed in the strata of Kap København about 2 million years ago broke the record for the oldest DNA ever sequenced.

**Figure:**
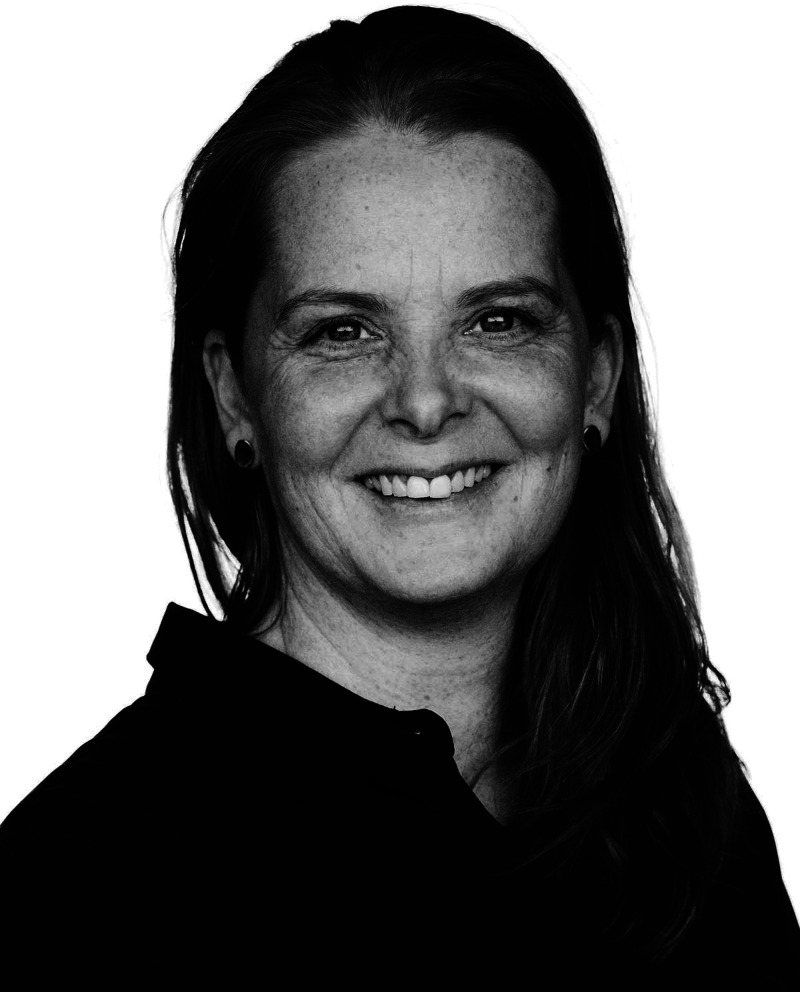
Credit: Thomas Frandsen/Villum Fonden.

But the crucial step—the recovery of the DNA from the minerals—did not come easy. Researchers had been working with the sample for years. “And they failed, and they failed. And at some point, it was like ‘This is cursed—we can’t do it,’ ” says Karina K. Sand, a molecular biogeochemist at the University of Copenhagen.

Sand’s knowledge of DNA-mineral interactions proved pivotal in guiding the team’s DNA extraction protocols. Her research centers on how minerals latch onto and preserve DNA, and she and her colleagues are continuing to explore how different minerals and environmental conditions affect the degradation of the DNA that minerals capture.

Carolyn Wilke spoke with Sand about her role in this work, the interplay between organic molecules and rock surfaces, and how that interplay could be part of evolution. This interview was edited for length and clarity.

## Tell me about the efforts to extract the 2-million-year-old DNA and why your expertise was needed.

At first, I wasn’t involved with that work. The researchers weren’t thinking about the nanolevel. They were thinking, “Well, you can’t see DNA on these samples.” But when you zoom in, you really can see the DNA, and you can see how tightly it is bonded and how it behaves on different mineral surfaces. I think for [Eske Willerslev, one of the project leaders], that opened up a new way of addressing this sample.

## How did your understanding of DNA-mineral interactions inform the team’s extraction protocol?

In some extraction protocols, the mineral portion is quickly discarded during the extraction process, as a lot of focus has been on biological materials, such as plant matter. But I was looking into what type of minerals there were in the deposit and the ability of those minerals to actually adsorb and retain DNA.

**Figure:**
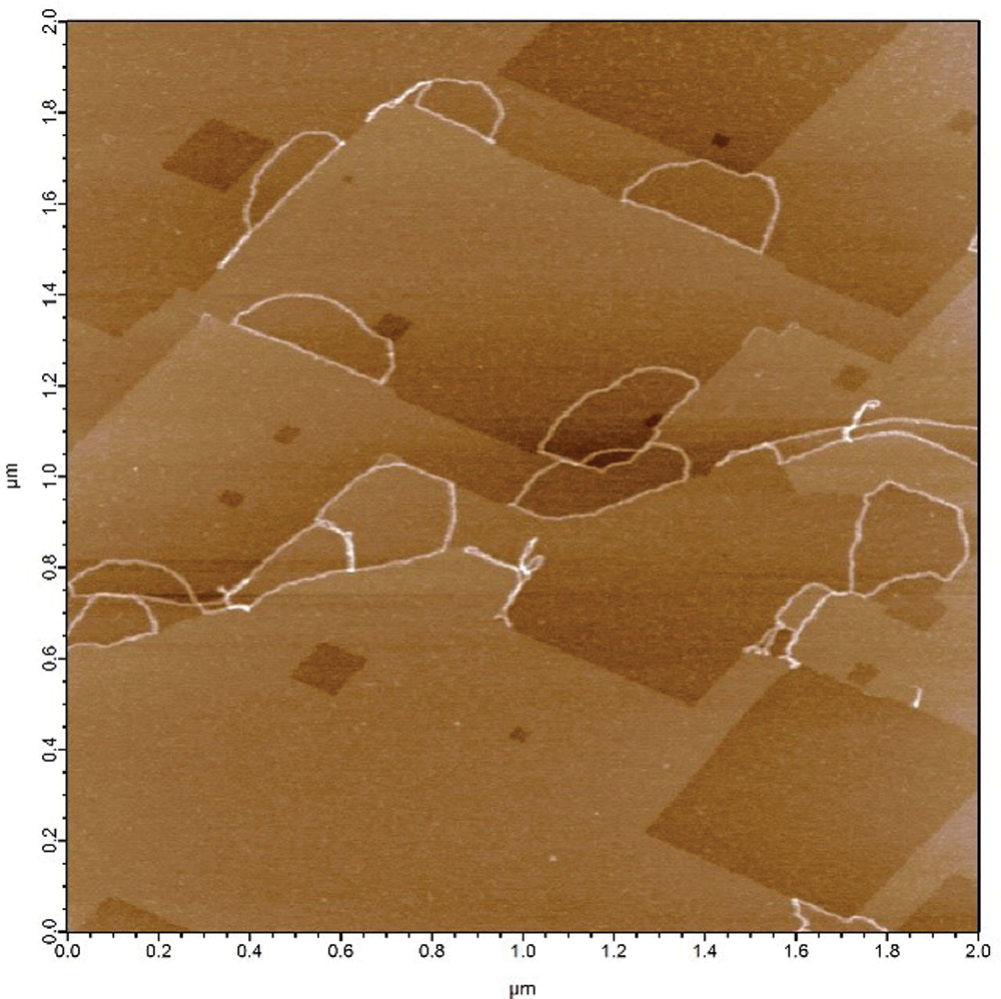
Using an atomic force microscope, researchers can see circular DNA (white lines) conforming to the angular surface of calcite. Credit: Léa Dieudonne.

We did a thorough mineralogic study where we quantified the minerals in the formation and did studies on the DNA adsorption capacities of each mineral. We also tested the extraction protocol in model studies—a range of minerals we deposited DNA onto—and found we could extract around 7% for some clays and much more for the nonclay silicates, such as quartz, feldspar, and pyroxene.

Using only existing degradation models, we shouldn’t have had any DNA that survived, but we saw that it had. We think that the DNA survived there because of the adsorption to mineral surfaces.

## How do minerals help preserve ancient DNA?

Minerals have active sites on their surfaces that have an affinity to bind things in solution. They collect a lot of stuff in the aquatic environment, and that includes extracellular DNA.

DNA’s backbone is made up of phosphate groups that are negatively charged in most environmental conditions. If you have a mineral that is positively charged, like carbonates, iron oxides, or clay edges, then the DNA will just adsorb directly, and that bond can be quite strong. When the DNA is stuck on the surface to the point where it doesn’t move, then many of the DNA functional groups aren’t available for chemical degradation, as they would be if the DNA were suspended in solution.

## What does the Kap København study mean for future research of ancient DNA preserved in sediments?

It opens up a whole new game. One of the big questions is to figure out biodiversity measures over time. So people could take a sediment core and link the biodiversity they observe to climatic changes, for instance. In my perspective, it would be quite relevant to look into samples from humid areas and areas where there’s higher temperature. People haven’t done that because we assume that DNA has been degraded.

The DNA can be really tightly adsorbed to certain types of minerals, which means it will survive longer. But it can be harder to extract. As we also showed in the Kap København paper, we’re not getting a lot of the DNA off some of the mineral surfaces, so we are currently testing an extraction protocol designed to extract more. I think there’s a huge potential.

**Figure:**
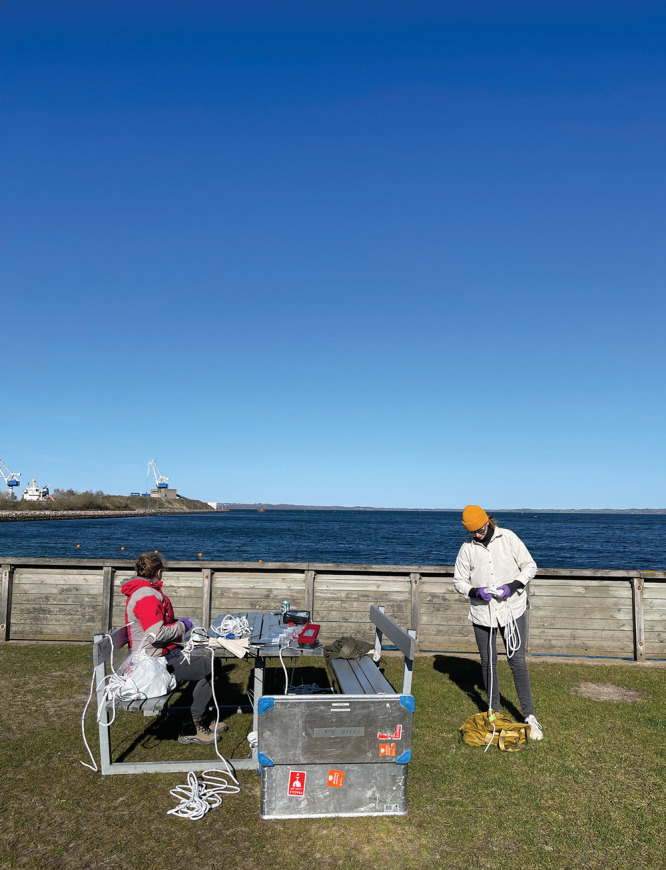
Karina Sand (right) and University of Copenhagen geoscientist Nicole Posth collect samples off the coast of Denmark for a study on marine microplastics and their role in the spread of antibiotic resistance in bacteria. Credit: Saghar Hendiani.

## What are you working on now?

My team also studies how life evolves. Bacteria can take up DNA from other bacteria through [a process called] horizontal gene transfer. Antibiotic resistance genes, for instance, are known to spread this way.

Normally, we think this happens by cell-to-cell contact, but DNA adsorbed to a mineral surface can also code for antibiotic resistance genes. If these genes are adsorbed to a mineral surface, they can be transported downstream to distant environments where microbes can actually pick up that DNA. We have done a lot of these studies on different mineral surfaces, and we can see the uptake frequency really depends on the DNA-mineral bond.

Microbes are able to get into most sedimentary systems, actually. So modern bacteria will be able to access a lot of ancient DNA and be able to take up lost traits. It’s an evolutionary pathway we haven’t really looked into.

## What was it like breaking this world record and being part of the team that sequenced the oldest DNA? How old do you think we can go?

I think it’s just kind of mind blowing that we can go back this far and map entire ecosystems. And when we have sections crossing time, we can also start looking at changes as a function of climate. It’s not a simple task, because the DNA would have different degradation rates depending on the different mineralogies, but there’s no doubt in my mind that we can go higher than 2 million years.

## Carolyn Wilke is a freelance contributor to

Chemical & Engineering News, *the independent news outlet of the American Chemical Society.*

